# Nanofat grafting under a split-thickness skin graft for problematic wound management

**DOI:** 10.1186/s40064-016-1808-2

**Published:** 2016-02-20

**Authors:** Cemal Alper Kemaloğlu

**Affiliations:** Department of Plastic, Reconstructive, and Aesthetic Surgery, Faculty of Medicine, Erciyes University, Talas, Kayseri Turkey

**Keywords:** Split-thickness skin graft, Fat graft, Stem cell, Extremity reconstruction

## Abstract

**Introduction:**

Obesity and certain medical disorders make the reconstruction of skin defects challenging. Different kind of procedure can be used for these defect, besides, skin grafting is one of the most common and simplest procedure. Fat grafting and stem cells which are located in the adipose tissue have been commonly used in plastic surgery for regeneration and rejuvenation purposes. To decrease graft failure rate we performed nanofat grafting under an autologous split-thickness skin graft in our patient who had a problematic wound.

**Case description:**

The case of a 35-year-old female patient with a traumatic skin defect on her left anterior crural region is described herein. After subsequent flap reconstruction, the result was disappointing and the defect size was widened. The defect was treated with combined grafting (nanofat grafting under an autologous split-thickness skin graft). At the 6 months follow-up assessment after combined grafting, the integrity of the skin graft was good with excellent pliability.

**Conclusions:**

Combined grafting for problematic wounds seems to be a useful technique for cases requiring reconstruction. The potential existence of stem cells may be responsible for the successful result in our patient.

## Background

Full-thickness skin defects frequently occur after trauma, vascular problems or tumor excision. Split-thickness skin grafts can be used to reconstruct these defects by applying them over the healthy recipient wound bed. Although split-thickness skin grafts can be easily used in lower limb reconstruction, they have higher failure and complication rates than those applied in other areas of the body since it is hard to keep the graft immobile. On the other hand, obese individuals are also at increased risk of wound complications including wound infection, dehiscence, hematoma, and seroma formation (Myers et al. 2007). Graft failure and chronic wounds in these patients are challenging problems and may also be expensive and time consuming to treat.

After the first reports on autologous fat grafting were published in the early twentieth century, it became popular in the plastic surgery armamentarium (Coleman 1995, 2001). Recent studies have demonstrated that the stromal-vascular cell fraction of adipose tissue represents a rich reservoir of regenerative precursor cells with proangiogenic capabilities (Zuk et al. 2001).

The term “nanofat grafting” was first used by Tonnard et al. and it can be used easily for skin rejuvenation purposes due to its small size and the fact that it contains stem cells (Tonnard et al. 2013).

The aim of this case report is to present an alternative method for managing chronic wounds of the lower limb by using nanofat grafting under an autologous split-thickness skin graft.

## Case presentation

**Fig. 1:**
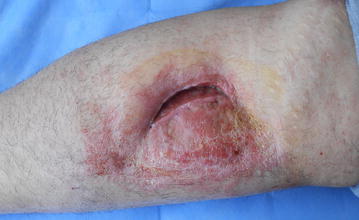
A 7 × 1.5 cm defect in the anterior crural region and previous skin grafted area are shown

**Fig. 2:**
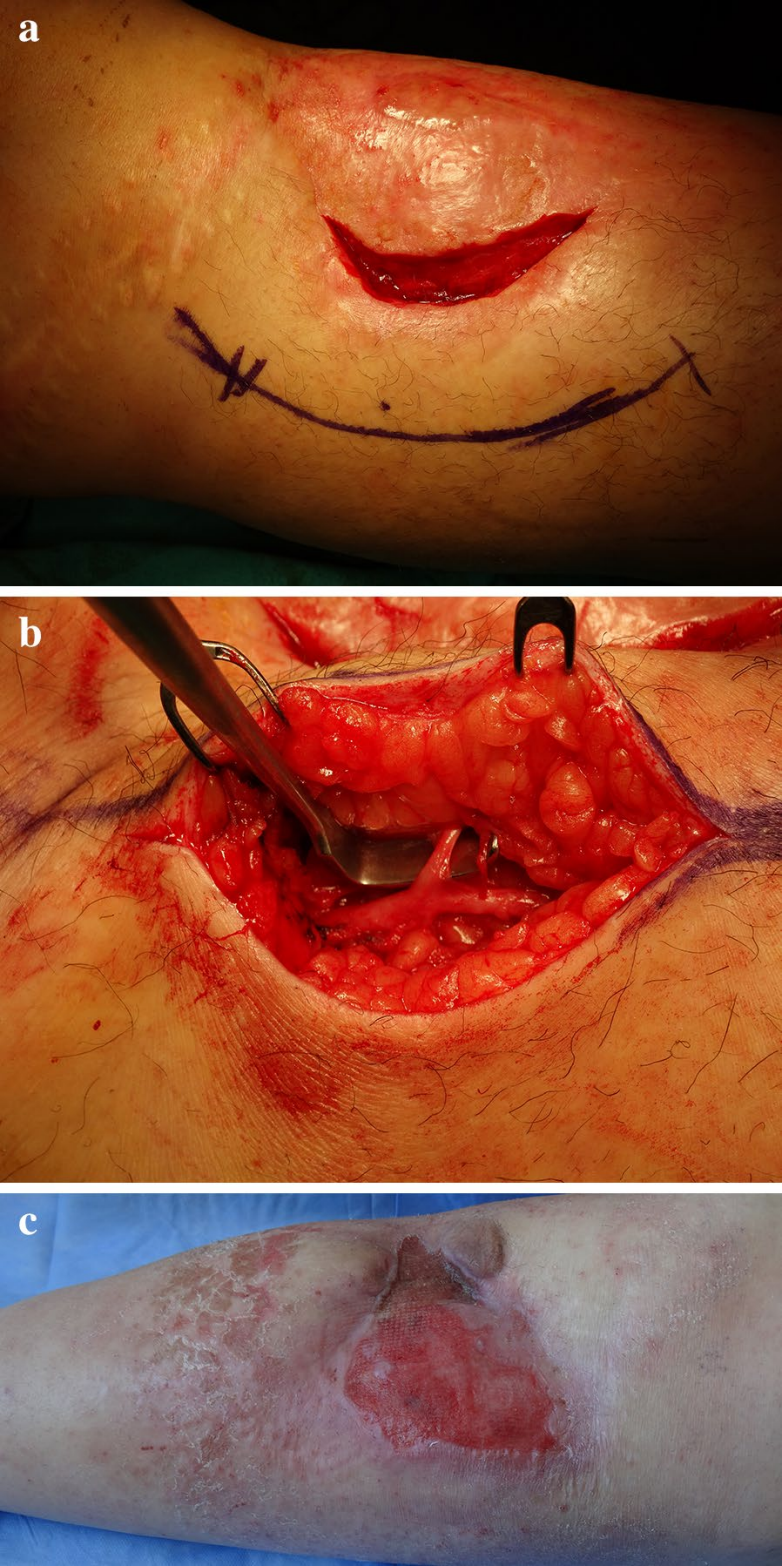
**a** Bipedicled flap with preserving the perforator was planned for reconstruction (**b**) preserved perforator is shown (**c**) 1 month after surgery the defect size extended to 12 × 7 cm

A 35-year-old female patient who had a full-thickness skin defect on her left anterior crural region due to trauma was referred to our unit in 2014. The patient had undergone an autologous skin graft procedure 3 months previously at another clinic before she came to us but partial graft failure had occurred 1 month after her first skin graft. In clinical examination the patient had a 7 × 1.5 cm defect in the anterior crural region just inferior to the patella; the body mass index (BMI) of the patient was 32 (Fig. 1). There were no other existing medical disorders. We performed a bipedicled flap with preserving perforator to reconstruct the defect but 1 month later the defect size had extended to 12 × 7 cm (Fig. 2). After these disappointing results we decided to treat the defect with nanofat grafting under the autologous split-thickness skin graft. Written informed consent was obtained from the patient and the operation was performed under general anesthesia.

## Surgical procedure and evaluation

**Fig. 3:**
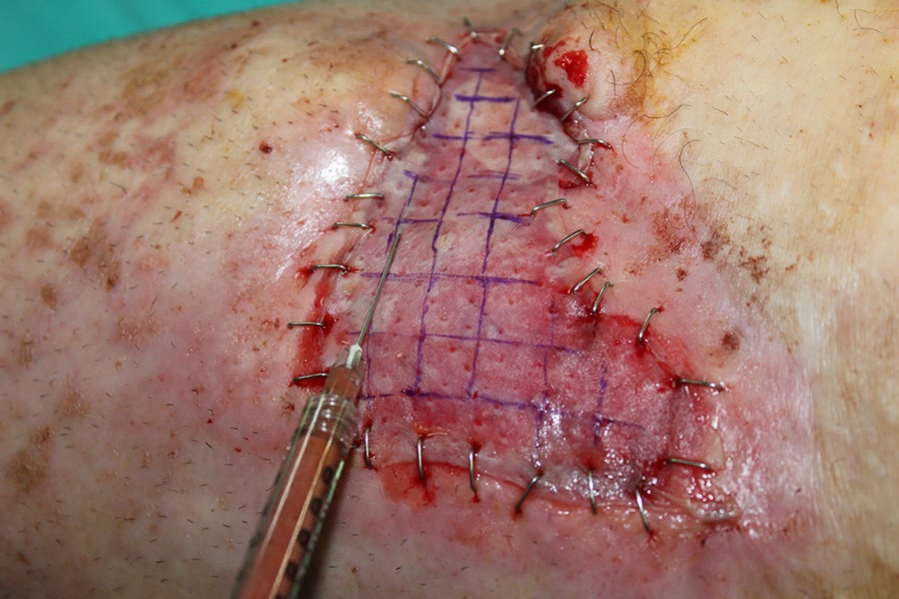
Nanofat graft was injected under the markings on the skin graft by 23-G cannula

The operation was performed by a senior surgeon (C.A.K.). Wound debridement was performed until all necrotic structures were removed and the viable tissues were reached. An autologous split-thickness skin graft (0.020 in. thick) was harvested from the posterior thigh using an electric dermatome (Integra^®^ Padgett^®^ Dermatome; Integra Inc., NJ, USA). The skin graft was placed on the wound and sutured by skin stapler (3 M™ Precise™ Vista Disposable Skin Stapler; 3 M Inc., Minneapolis, USA). Then, 1 cm length squares were marked on the skin graft to determine where the nanofat graft was to be injected. In order to achieve skin graft viability by diffusion, the fat graft was not injected under the whole skin graft. A few small holes were created in the middle of the squares on the skin graft by a no.11 blade to prevent hematoma under the graft. After the infiltration of modified Klein solution (lidocaine 800 mg/liter and adrenaline 1:1,000,000) into the lower abdomen, fat grafts were harvested by means of a 3-mm multiport harvesting cannula with side holes of 1-mm diameter. The harvesting cannula was attached to a 10-mL syringe and the plunger of the syringe was pulled back to create adequate negative pressure. Upon completion of the fat harvest into the 10-mL syringe, the fat grafts were transferred from the syringe to a blood collection tube. They were then placed in a sterilized centrifuge rotor and spun at 3000 rpm for 3 min to separate the components of the tissue. The oil on the surface was decanted and the middle layer was aspirated by a 10-mL syringe. To obtain the nanofat graft, according to the report of Tonnard et al., emulsification of the fat was achieved by shifting the fat between two 10-cc syringes connected to each other by a female-to-female Luer-Lock connector (Tonnard et al. 2013). In this way the microfat lipoaspirate was processed into nanofat. After 30 passes, the fat became liquid and this was filtered over a sterile nylon cloth so as to remove the connective tissue and the effluent (nanofat graft) was collected. Then the nanofat graft was transferred to 1-mL syringes. It was injected under the markings on the skin graft by a 23-G cannula. Then, an ointment-impregnated gauze and tie-over dressing was applied on the skin graft for 6 days (Fig. 3). The patient was followed up for 6 months. The results were photographically documented at each clinical visit.

**Fig. 4:**
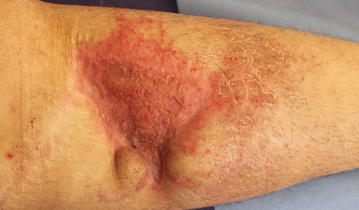
Appearance of the defect 6 months after surgery. The skin graft healed uneventfully with good pliability

Clinical assessment was performed 6 days, 2 weeks, 1, 3 and 6 months after combined grafting (nanofat grafting with autologous skin grafting). During the follow-up period no wound or donor site complication was observed, such as graft detachment, graft hematoma, graft losses or hematoma and infection in the donor area. The defect was totally reconstructed by combined grafting uneventfully (Fig. 4). Moreover, the pliability and vascularity of the skin graft was also satisfactory. During the 6-month follow-up period such benefits remained stable without recurrence of skin integrity problems.

## Discussion

Lower extremity reconstruction can be complicated for both patients and surgeons. Unlike the head and neck, the lower limb is essential for mobilization and, as a result, movement can cause shear forces on the repair. Increased hydrostatic forces in the lower limb during mobilization may lead to hematoma or seroma formation. If there is no exposed bone, tendon or vital tissues exist, various techniques can be used like secondary healing, skin grafting or flap surgery. Although secondary healing is the simplest method, it can only be used for small defects. In defects which have a healthy wound bed, skin grafting is the most convenient technique for reconstruction.

A higher BMI has repeatedly been correlated with increased incidence of surgical complications, including atelectasis, thrombophlebitis, mortality, wound infection, flap necrosis and wound separation (Pitanguy et al. 2000; Matory et al. 1994; Selber et al. 2006). Thus, lower extremity reconstruction in obese patients can be challenging due to the possibility of potential wound healing problems. Although the use of a vacuum assisted closure device (VAC) on split-thickness skin grafts is associated with improved graft survival (Scherer et al. 2002), there is no evidence about the benefits of combined VAC therapy with skin graft in obese patients.

After the first adipose-derived stem cells were discovered in 2001, they have been used for various purposes, including the regeneration of missing tissues and rejuvenation (Rigotti et al. 2009; Rehman et al. 2004). Adipose-derived stem cells have an extensive proliferative capacity and the ability to differentiate into mesoderm, ectoderm, and endoderm lineages (Brzoska et al. 2007). To isolate stem cells, the stromal vascular fraction has to be separated from the adipocytes. However, isolating the stromal vascular fraction from the adipocytes before injection in routine clinical cases would be time consuming, complicated, and expensive. Therefore obtaining nanofat grafting is the simplest method to achieve adipose-derived stem cells and performing the injection with small cannulas in very small amounts is another advantage of this procedure. Although the regeneration properties of adipose-derived stem cells in clinical usage have been reported for radiodermatitis, atrophic scars, chronic ulcerations and antiageing therapy, this case is the first in the literature to report nanofat graft use for the management of a wound healing problem combined with skin grafting (Rigotti et al. 2007; Sarfati et al. 2011).

Due to the amount of dermis, full-thickness skin grafts provide better scar quality and less contraction. However, large defects can not be reconstructed by full-thickness skin graft because of limited donor area. Replacement of all the missing parts of a wound is essential to achieve the best scar and functional results. Therefore, the advantages of using a split-thickness skin graft were combined with nanofat grafting to obtain subdermal fat tissue and the stimulation of collagen by potential stem cells which are located in a nanofat graft (Tonnard et al. 2013).

To the best of our knowledge, the present study is the first to investigate the use of autologous split-thickness skin grafting with nanofat grafting. Because of previous skin graft failure history, a combined grafting technique was tried in our patient to provide better outcomes without increasing the complications. Stem cells can be differentiated to other cells in 2 weeks (Zuk et al. 2001). The probable mechanism of increased graft take, such as was observed in our obese patient, may have resulted from the stimulation of collagen and endothelial cells by stem cells. Endothelial cell stimulation promotes angiogenesis and these properties are very important not in the acute but in the vascular ingrowth phases of graft healing (Rigotti et al. 2007).

Regardless of the fact that adipose-derived stem cells have a function in combined grafting, molecular and immunohistochemical investigations should be conducted in future clinical studies. They may also be suitable for future use in combined grafting for difficult wounds, such as wound beds with poor granulation or small exposed tendons without paratenon.

## Conclusions

Wound healing problems are common in obese patients. Split -thickness skin grafting is the simplest technique for the treatment of wounds which have good granulation tissue. Nowadays, adipose- derived stem cells are widely used in plastic surgery practice. Combining a skin graft with nanofat grafting may be an alternative for difficult cases. Further investigation which provides histopathologic and immunological evidence is necessary to support our findings.
